# Aberrant lipid accumulation in the mouse visceral yolk sac resulting from maternal diabetes and obesity

**DOI:** 10.3389/fcell.2023.1073807

**Published:** 2023-03-01

**Authors:** Man Zhang, J. Michael Salbaum, Sydney Jones, David Burk, Claudia Kappen

**Affiliations:** ^1^ Developmental Biology, Baton Rouge, LA, United States; ^2^ Regulation of Gene Expression, Baton Rouge, LA, United States; ^3^ Cell Biology and Bioimaging Core, Baton Rouge, LA, United States

**Keywords:** lipid droplet, lipid transporter, lipid deficiency, neural tube defect, hyperglycemic, hyperlipidemia, hyperglycemia

## Abstract

Maternal diabetes and obesity in pregnancy are well-known risk factors for structural birth defects, including neural tube defects and congenital heart defects. Progeny from affected pregnancies are also predisposed to developing cardiometabolic disease in later life. Based upon *in vitro* embryo cultures of rat embryos, it was postulated that nutrient uptake by the yolk sac is deficient in diabetic pregnancies. In contrast, using two independent mouse models of maternal diabetes, and a high-fat diet-feeding model of maternal obesity, we observed excessive lipid accumulation at 8.5 days in the yolk sac. The numbers as well as sizes of intracellular lipid droplets were increased in yolk sacs of embryos from diabetic and obese pregnancies. Maternal metabolic disease did not affect expression of lipid transporter proteins, including ApoA1, ApoB and SR-B1, consistent with our earlier report that expression of glucose and fatty acid transporter genes was also unchanged in diabetic pregnancy-derived yolk sacs. Colocalization of lipid droplets with lysosomes was significantly reduced in the yolk sacs from diabetic and obese pregnancies compared to yolk sacs from normal pregnancies. We therefore conclude that processing of lipids is defective in pregnancies affected by maternal metabolic disease, which may lead to reduced availability of lipids to the developing embryo. The possible implications of insufficient supply of lipids -and potentially of other nutrients-to the embryos experiencing adverse pregnancy conditions are discussed.

## Introduction

Maternal diabetes and maternal obesity during pregnancy are known risk factors for structural birth defects, such as cardiovascular malformations and neural tube defects (NTDs) (Mills, 1982; Martinez-Frias, 1994; Martinez-Frias et al., 2005; Helle and Priest, 2020). The causative mechanisms underlying the elevated risk conferred in these pregnancies have not been identified, although it is generally accepted that nutrient excess, such as in hyperglycemia of the mother, plays a critical role.

During the formation of heart and neural tube, the embryo receives nutrients through the yolk sac, which serves as the ‘primitive placenta’ during the early post-implantation development (Zohn and Sarkar, 2010). The yolk sac tissues are comprised of the outer parietal yolk sac and the inner visceral yolk sac. The visceral yolk sac consists of a layer of endodermal cells facing the yolk sac cavity and underlying mesodermal cells that face the embryonic cavity. The visceral yolk sac thus functions as the site for the uptake and processing of nutrients by endocytic cells in the endodermal layer, while the transport of nutrients to the embryo is accomplished by vessels in the mesodermal layer that form from a common precursor of endothelial and hematopoietic progenitor cells (Zohn and Sarkar, 2010).

Prior studies on rat conceptuses cultured *in vitro* have provided evidence that exposure to high glucose concentrations was associated with yolk sac morphological abnormalities (Pinter et al., 1986b), reduced uptake of horseradish peroxidase (as an indicator for endocytosis) (Reece et al., 1989), reduced fatty acid uptake from the medium (Pinter et al., 1988) and decreased lipid droplet content (Reece et al., 1994). Several of these outcomes could be alleviated by the addition of arachidonic acid (Pinter et al., 1986a; Pinter et al., 1988). These authors also reported that vessel coverage of the yolk sac surface was reduced by excessive glucose in the culture medium (Pinter et al., 1986b). Culture of mouse conceptuses yielded confirming observations of vasculopathy (Pinter et al., 1999), which could be ameliorated by addition of an external nitric oxide donor (Nath et al., 2004). Although some similarities between yolk sacs freshly isolated from diabetic pregnant dams and cultured high-glucose-exposed specimens were observed in both the rat and mouse models (Reece et al., 1994; Pinter et al., 1999; Nath et al., 2004), the culture of post-implantation conceptuses *in vitro* is confounded by addition of adult serum, and an excessively high oxygen atmosphere. In contrast, implantation sites *in vivo* are in hypoxic conditions, which stimulate and are required for vasculogenesis (Ryan et al., 1998).

In the present study, we therefore sought to re-appraise the *in vivo* manifestation of yolk sac abnormalities in two mouse models of diabetic pregnancy, spontaneously occurring diabetes in the non-obese diabetic (NOD) mouse strain (Leiter, 1989; Atkinson and Leiter, 1999), and Streptozotocin-induced diabetes in the FVB/N mouse strain (Pani et al., 2002; Qi et al., 2005; Pavlinkova et al., 2009). In order to model the hyperlipidemia that is characteristically associated with diabetic pregnancy, we also investigated pregnancies in FVB/N mice fed a high-fat-high-sucrose diet for 4 weeks prior to mating (Salbaum and Kappen, 2012). The specific focus on yolk sac was prompted by our earlier findings of layer-specific expression of glucose and fatty acid transporters in visceral yolk sac endodermal and mesodermal cells (Kappen et al., 2022). Since expression of these transporters at the mRNA level was unaffected by exposure to maternal diabetes in decidua, embryos, and yolk sacs, we hypothesized that abnormalities in nutrient uptake, processing and/or transport caused by the exposure should be evident at the functional level.

## Materials and methods

### Animals

All animal experiments were performed with prior approval of the Pennington Biomedical Research Center Institutional Animal Care and Use Committee (IACUC) in accordance with the “Guide for the care and use of laboratory animals” of the United States National Institutes of Health. The experimental model consisted of female mice of the FVB strain and the Non-Obese Diabetic (NOD) strain. Mice of the FVB inbred strain were obtained from Charles River Laboratories at the age of 5–6 weeks, and NOD strain animals were obtained from The Jackson Laboratories at the age of 7–8 weeks. Mice were accommodated to the animal facility for 1 week before experimentation.

Two diabetic mouse models were used in our experimentation. Diabetes in FVB strain was induced by three i. p. Injections within a week of Streptozotocin at a dose of 100 mg/kg body weight, as previously described (Kappen et al., 2011, 2012). Females whose blood glucose levels exceeded 250 mg/dl were considered diabetic and were set up for mating no earlier than 7 days after the last STZ injection. The NOD strain of mice is an established model for human type I diabetes (Leiter, 1989; Leiter, 2005), in which individuals turn diabetic spontaneously. Approximately 60% of females of this strain exhibit profound hyperglycemia between 12–17 weeks of age, so that normoglycemic non-diabetic females of comparable age can be used as controls. When blood glucose levels exceeded 250 mg/dl, females were designated hyperglycemic and were mated to normoglycemic chow-fed males of the same strain, respectively.

The maternal obesity model was set up by using the FVB strain: females were fed with high-fat-high-sucrose diet (D12331, Research Diets, New Brunswick, NJ) for 4 weeks prior to mating, a regimen that we previously showed produces about 25% adiposity while preserving fertility (Salbaum and Kappen, 2012). The diet consists of 16.4 kcal% protein, 25.5 kcal% carbohydrate (sucrose content is 18.4% of total weight), 58.0 kcal% fat, and is replete for minerals, Vitamins and micronutrients and was fed through pregnancy until sacrifice. Diabetic and high-fat-diet-fed females were bred to normoglycemic males of the respective strain that had been fed regular mouse chow diet (LabDiet 5001, LabDiet, St. Louis, MO). Vaginal plugs were checked in the morning after mating, and the day of detection of a plug was counted as day 0.5 of gestation.

### Bodipy staining and three-dimensional image analysis

Conceptuses at E7.5 were isolated from normoglycemic and diabetic NOD pregnancies, and the parietal yolk sacs were removed. Staining with Bodipy was done as described below, with counterstain for Actin filaments (F-actin) by Phalloidin (Abcam, Waltham, MA). Conceptuses at E8.5 were recovered from normal pregnancies, diabetic pregnancies, and high-fat-diet (HFD) pregnancies, and those yolk sac tissues that could be dissected away from the embryo were freshly isolated. The developmental stage of cognate embryos was recorded based upon the. number of somite pairs, and somite stages were variable between individuals from a given litter, as expected. In the aggregate, the distribution of stages between the groups of normal samples was comparable to that of samples from diabetic and of high-fat diet-fed pregnancies within the same strain, respectively.

Following fixation in 4% PFA for 30 min, yolk sacs were washed 3 times in phosphate-buffered saline (PBS) for 10 min. Bodipy (Invitrogen, Waltham, MA, 1:1000) was diluted in PBS at a concentration of 1 mg/ml and applied to the yolk sacs at 37°C for 15 min, followed by the incubation of 4’,6-diamidino-2-phenylindole (DAPI; 1:1000 in PBS) at room temperature for 5 min. Serial Z-stack fluorescence images from each sample were obtained by a Leica SP5 spectral scanning confocal microscope (Leica, Wetzlar, Germany). Imaris software was employed to reconstruct three-dimensional images and quantify the individual three-dimensional lipid droplets and their volumes in yolk sac samples. Specifically, contour surfaces were created for both Bodipy and DAPI fluorescence, individual fluorescence particles were identified and their volumes calculated, respectively. Then the fluorescence intensities were quantified by summing up of all the particle volumes, as implemented in the Imaris package. The relative fluorescence volumes were calculated as the ratio of the Bodipy and DAPI total volumes.

### Immunohistochemistry (IHC) staining

Paraffin embedded yolk sac sections were used for IHC staining. For deparaffinization, slides were heated for 60 min at 65°C, followed by three treatments with 100% xylene, then two times with 100% ethanol, one time with 95% ethanol and 70% ethanol, for 5 min per step. For antigen retrieval, slides were incubated with 0.01M citrate buffer (pH 6.0) at 90°C in a pressure cooker for 20 min. Then, the slides were treated with a 3% H_2_O_2_ solution for 15 min to saturate endogenous peroxidases. After blocking with horse serum for 1 h at room temperature, slides were incubated overnight at 4°C with primary rabbit antibodies against ApoA1 (Thermo Fisher Scientific, Waltham, MA, 1:200), or ApoB (Abcam, Cambridge, United Kingdom, 1:200), or SR-B1 (Abcam, Cambridge, United Kingdom, 1:100). On the second day, after washing three times with PBS, the slides were incubated with HRP micropolymer-conjugated horse anti-rabbit IgG (Vector Laboratories, Burlingame, CA) at room temperature for 1 h. Slides were then washed with PBS and incubated with DAB, an HRP substrate (Vector Laboratories, Burlingame, CA) for 2 min, followed by dehydration, including one time of 70% ethanol and 80% ethanol, two times of 95% ethanol and 100% ethanol, and 100% xylene for 10 min. The slides were coverslipped with permanent mounting medium (Vector Laboratories, Burlingame, CA) and imaged under bright field illumination on an optical microscope (Zeiss, Oberkochen, Germany).

### Immunofluorescence (IF) staining and quantification

Paraffin embedded tissue sections were used for IF staining. After deparaffinization and antigen retrieval procedures (described in the IHC staining method above), slides were incubated in 5% donkey serum in 1% BSA for 60 min for blocking, then washed in PBS three times. Slides were incubated overnight at 4°C with primary rabbit antibodies against ApoA1 (Thermo Fisher Scientific, Waltham, MA, at 1:100 dilution), ApoB (Abcam, Cambridge, United Kingdom, at 1:100 dilution), SR-B1 (Abcam, Cambridge, United Kingdom, at 1:100 dilution) and Folate receptor α (Thermo Fisher Scientific, Waltham, MA, at 1:50 dilution). The following day, slides were washed three times with PBS and incubated at room temperature for 60 min with donkey anti-rabbit Alexa Fluor 488-conjugated (Thermo Fisher Scientific, Waltham, MA, 1:500) and donkey anti-sheep Alexa Fluor 555-conjugated secondary antibodies (Thermo Fisher Scientific, Waltham, MA, 1:200). Slides were then washed three times in PBS and covered with antifade mounting medium with DAPI (Vector Laboratories, Burlingame, CA). Slides were stored in the dark at 4°C until imaged with the Sp5 confocal microscope (Leica, Wetzlar, Germany). Total fluorescence intensities were quantified for lipid transporter proteins and DAPI by ImageJ. The relative fluorescence intensities were calculated as the ratio of total fluorescence intensity of these lipid transporter proteins over DAPI.

### LysoTracker staining and colocalization analysis

Fresh yolk sacs were collected from normal, diabetic and obese pregnancies at embryonic day 8.5 (E8.5). LysoTracker™ Deep Red (Thermo Fisher Scientific, Waltham, MA, 1:10,000) and Bodipy (Invitrogen, Waltham, MA, 1:1000) were diluted in Opti-MEM (Gibco, Waltham, MA) and applied to the yolk sacs at 37°C for 25 min. Following washing with Opti-MEM for 5 min three times, the yolk sacs were immobilized in 35 mm glass bottom dishes (Mattek, Ashland, MA) by a method modified from a previous study (Aoyama et al., 2012). 0.2% gellan gum (Sigma, St. Louis, MO) was mixed in 40% glycerol in PBS and microwaved for 30 s 500–1000 μl of hot gellan gum solution was poured into a 35 mm glass bottom dish (Mattek, Ashland, MA). The dishes were stored in the refrigerator to further harden after solidification. A small cut was made in the middle of the gellan gum gel using a syringe needle, and the yolk sacs were placed beneath the cut gel. Immobilized yolk sac tissue stained with Lysotracker and Bodipy was imaged by a Leica SP5 spectral scanning confocal microscope (Leica, Wetzlar, Germany). The ImageJ plugin “Colocalization Image Creator” was used for quantification of colocalization of both stains at default settings. Areas where Bodipy staining was colocalized with LysoTracker staining, and the individual lysosome areas were identified and quantified by ImageJ. A colocalization area fraction was calculated by the ratio of colocalized regions and the corresponding lysosome region.

### Next-generation sequencing (RNAseq.)

The technical details of these experiments are described elsewhere (J.M. Salbaum, K. Stone, C. Kruger, and C. Kappen, manuscript submitted); the results have been deposited in the Gene Expression Omnibus database under accession number GSE197396. Briefly, yolk sacs were isolated from E8.5 pregnant dams of the NOD mouse strain that were either normoglycemic or diabetic. Individual samples were processed for RNAseq., sequence read counts were normalized to the size of each sequencing library, and analysis was performed using DESeq2. *p*-values for comparison of averages between control and experimental group were adjusted for multiple comparisons within the entire dataset, as implemented in DESeq2. For this manuscript, the gene ontology (GO) annotations of this dataset were queried with the search terms lipid, droplet, lipoprotein, lysosome, lipolysis, autophagy, Lrp, Snx, Perilipin, Cubilin, Enpp, and FolR1, and genes with low abundance (normalized read count below 50) were eliminated.

### Statistical analysis

Data are representative of at least three independent experiments unless otherwise specified. Quantitative data are expressed as average ±SD; samples from the same pregnancy were first normalized to each pregnancy, before averages were tallied by experimental group. Student’s t-test was used to evaluate statistical significance between two groups, based upon two-tailed distributions and assumption of unequal variances. All statistical analyses were done using Graph Pad Prism software, and the criterion for significance was considered *p* < 0.05.

## Results

**FIGURE 1:**
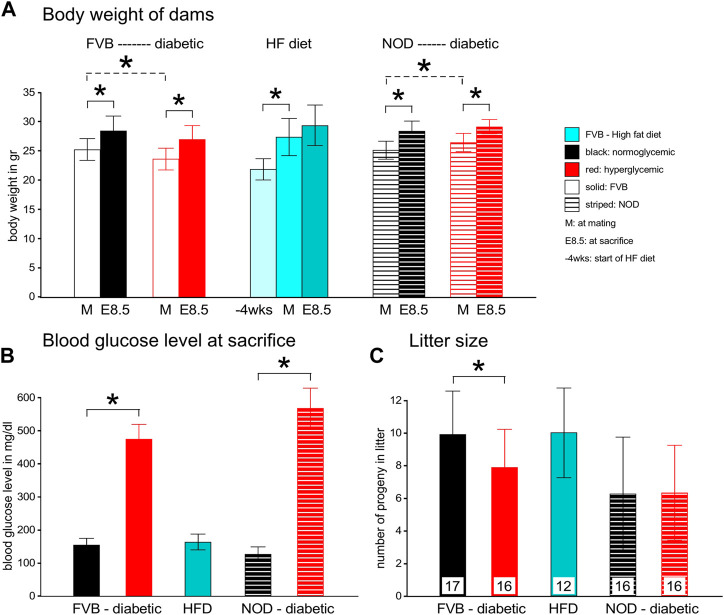
Maternal parameters in two experimental models of diabetic pregnancy and a mouse model of obesity in pregnancy. Panel **A:** FVB females entered the experiments at the age of 10–12 weeks, after diabetes induction by STZ, whereas NOD females were used between the ages of 12–17 weeks, depending on development of diabetes. FVB females with high-fat diet feeding entered the feeding stage at ∼8 weeks of age, with matings performed at ∼12 weeks of age. Maternal body weights were recorded at entry into the feeding phase, at mating and at sacrifice at gestational day E8.5. Diabetic cohorts are labeled in red, FVB animals are represented by solid bars, NOD mice by striped bars. Panel **B**: Blood glucose levels were measured in FVB dams before and after STZ-induction, and at mating and sacrifice. In NOD females, blood glucose levels were monitored weekly after the age of 12 weeks. Females were declared diabetic and mated when their blood glucose levels exceeded 250 mg/dl; their glucose levels increased further throughout the pregnancy. Glucose measurements on HFD-fed females were taken at mating and sacrifice, and remained normal throughout the pregnancy. Panel **C**: Litter sizes were reduced in FVB diabetic females, and remained unaffected by HFD and in the NOD strain. Number of pregnant females/group: FVB normoglycemic n = 17; FVB diabetic n = 16; FVB HFD: n = 12; NOD normoglycemic n = 16; NOD diabetic n = 16.

Morphometric parameters of the mouse models employed in these studies are depicted in Figure 1. Body weight was recorded at the time of inspection for a copulation plug (days 0.5 of gestation, Figure 1, Panel A, light bars), and at the time of sacrifice at 8.5 days of gestation (E8.5, filled bars). Diabetic FVB females started the pregnancy slightly lighter (compare open red bar to open black bar, *p* = 0.017), but gained weight to the extent that the difference was not statistically significant at the time of euthanasia. FVB females fed a high-fat-high-sucrose diet for 4 weeks gained more than 5 g body weight on average (compare dark to light turquoise bars; ranging from 2.7–9.3 g gained individually) up to the time of mating compared to the chow-fed FVB controls; they gained another 2 g on average during the pregnancy; no single individual lost weight. The NOD females also gained weight during pregnancy, and while the diabetic group started the pregnancy slightly heavier than the normoglycemic controls (*p* = 0.023), the differences were no longer statistically significant at the time of euthanasia.

Consistent with our definition of hyperglycemia beyond 250 mg/dl blood glucose content, both the FVB and NOD diabetic groups at E8.5 had high blood glucose levels that even exceeded the upper sensitivity of the glucometer in 3 NOD females (Figure 1, Panel B). High-fat diet-fed females were normoglycemic throughout the experiment. Diabetic FVB females had significantly smaller litter sizes compared to normoglycemic controls (*p* = 0.028), which was not observed in the NOD strain (Figure 1, Panel C), which had generally smaller litters. Notably, high-fat-diet-feeding had no effect on litter size.

### Excessive lipid accumulation in yolk sacs of pregnancies affected by type I diabetes

**FIGURE 2:**
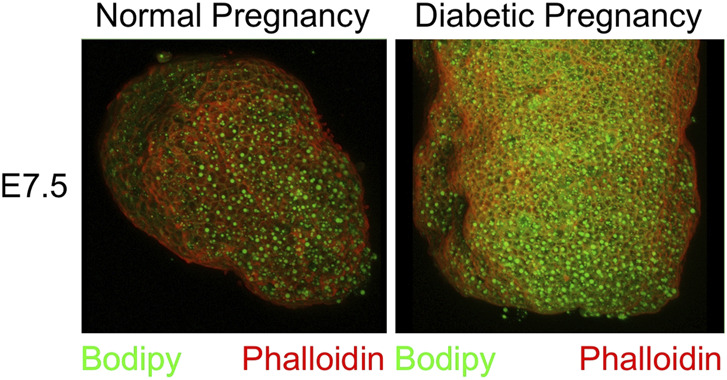
Accumulation of lipid droplets in yolk sac of diabetes-exposed embryos. Conceptuses were isolated from normoglycemic and diabetic pregnant NOD dams at gestational day E7.5, and dissected free of the parietal yolk sac. They were stained with Phalloidin and Bodipy. The conceptus from a diabetic pregnancy displays more lipid droplets in the visceral yolk sac.

Upon dissection of E7.5 conceptuses under the microscope (for other experiments), we observed greater deflection of light and reduced translucency when the samples came from a diabetic pregnancy. This prompted us to stain whole conceptuses for lipids by using Bodipy, and for actin as a general marker for cells by using Phalloidin, after removal of the parietal yolk sac. In the examples shown in Figure 2, greater intensity of Bodipy fluorescence was evident in the sample from a diabetic NOD pregnancy when compared to a sample from a normal NOD female at E7.5, suggesting that there was excessive accumulation of lipids in the yolk sac in the diabetic condition.

**FIGURE 3:**
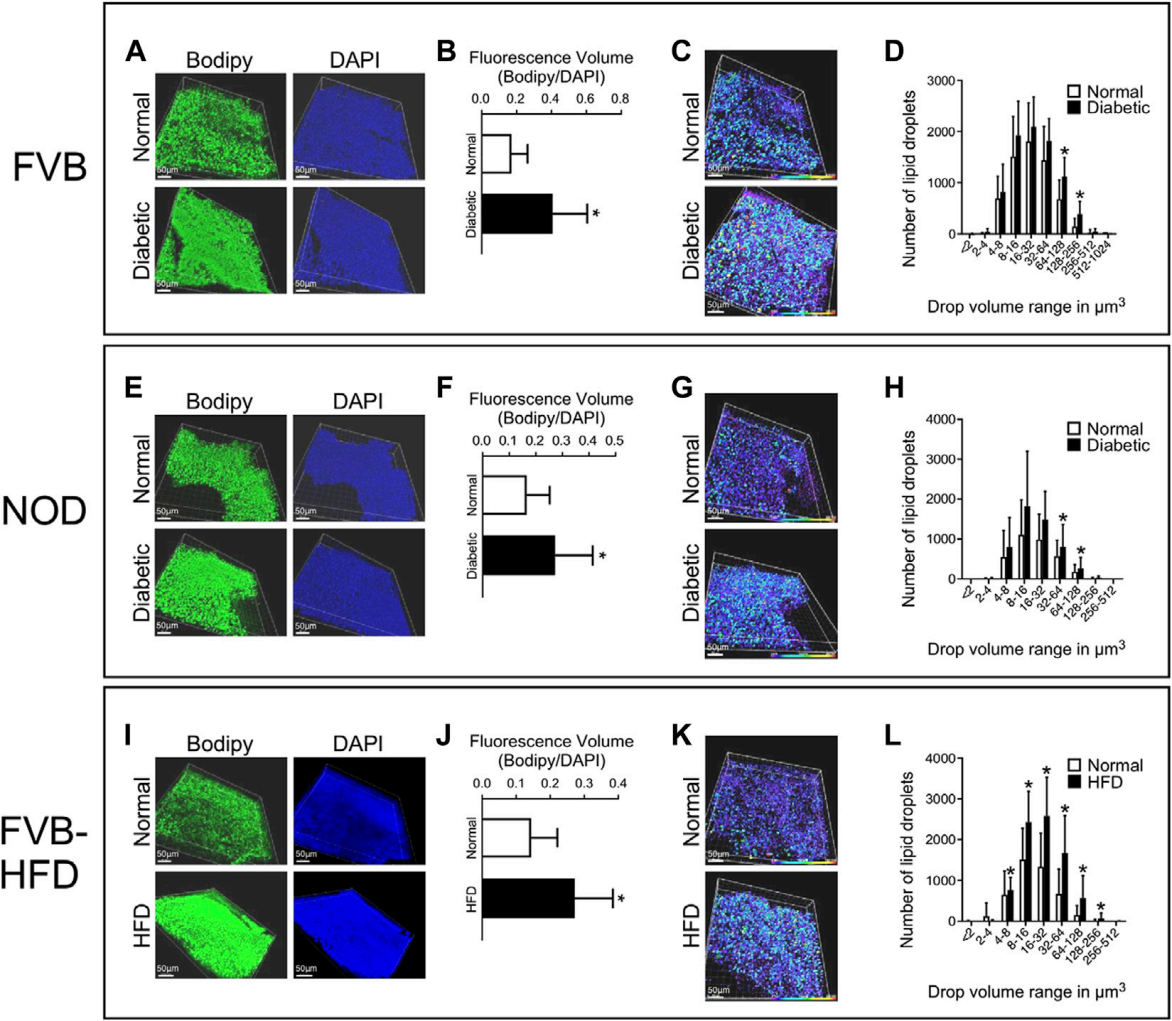
Excess lipid accumulation in yolk sacs from diabetic and obese pregnancies. Confocal imaging was performed on E8.5 yolk sacs stained with Bodipy (lipids) and DAPI (DNA) for all three experimental models. Panels **A**, **E**, and **I**: Three-dimensional renderings of signals for lipids and DNA. Panels **B**, **F**, and **J**: Voxels detected in each channel were quantified, and the ratio of lipid over DAPI voxels was calculated. This ratio was significantly higher in visceral yolk sac from diabetic FVB and NOD pregnancies, and in pregnancies where the dam was fed high-fat diet. Panels **C**, **G** and **K**: Volumes of lipid droplets were pseudo-colored for imaging according to size, following the scale at the right bottom of each image, purple-blue = smaller, yellow-red = larger volume. Yolk sacs from diabetic pregnancies and from dams fed HFD display appreciably more droplets with colors representing larger volume ranges. Panels **D**, **H** and **L**: Quantification of the numbers of lipid droplets within a given size range; note that the scale of the *X*-axis is logarithmic (log2), and that *X* and *Y* axis scales differ when comparing **D** to **H** and **L**. Frequencies of occurrence are shifted towards lipid droplets of larger volumes in yolk sacs from diabetic FVB and NOD dams, and HFD pregnancies. Sample size: FVB Normal = 25 samples representing 14 yolk sacs from 4 pregnancies, FVB diabetic = 43 samples representing 21 yolk sacs from 7 pregnancies; NOD Normal = 26 samples representing 14 yolk sacs from 5 pregnancies, NOD diabetic = 36 samples representing 25 yolk sacs from 9 pregnancies; FVB Normal = 42 samples representing 14 yolk sacs from 4 pregnancies, FVB-HFD = 68 samples representing 24 yolk sacs from 7 pregnancies. Data were averaged for each yolk sac (where multiple fragments were analyzed), and then averaged over all yolk sacs from a given pregnancy, after which averages were calculated for all pregnancies in the respective experimental group; Averages are presented as mean ± SD. Asterisks indicate *p* < 0.05, when comparing experimental and control groups using two-tailed t-tests.

In order to quantify lipid abundance, we chose to investigate conceptuses at the E8.5 time point, which facilitates dissection and provides larger tissue samples. Visceral yolk sacs were isolated by microdissection, and stained with Bodipy (lipid droplets) and DAPI (nuclei). Three-dimensional renderings were created by Imaris software from serial Z-stack fluorescence images of each sample. The individual fluorescence particles from both Bodipy and DAPI staining were identified and quantified in volume. The total fluorescence volume was calculated by summing up of all particle volumes for a given fluorescence channel. The fraction of total fluorescence volume of Bodipy over that of DAPI represents the lipid accumulation in each sample. Fluorescence images are shown in Figure 3 Panel A, and quantification data in Figure 3 Panel B. There was significantly increased lipid accumulation in yolk sacs from FVB-STZ diabetic pregnancies, compared to yolk sacs from normal FVB pregnancies. In addition, there was a greater number of lipid droplets, and the sizes of lipid droplets were larger in diabetic conditions, as shown in Figure 3 Panel C, where the size of lipid droplets is color-coded along the spectrum shown at the bottom of each image. Frequencies of occurrence of each droplet size range were quantified, and droplets of larger sizes than normal were more abundant in diabetic pregnancies (Figure 3 Panel D). Similarly, we found elevated lipid accumulation in NOD-diabetic pregnancies, again with increased droplet size and number (Figure 3 Panels E–H). The results demonstrate, in two mouse models of diabetic pregnancy, that in diabetic conditions the conceptus contains excess accumulation of lipids in the visceral yolk sac, characterized by increased number and volume of lipid droplets.

### Excessive lipid accumulation in yolk sacs of pregnancies affected by obesity

Applying the same experimental approach, we also observed increased lipid accumulation in yolk sacs from FVB pregnancies where the dam was fed a high-fat-high-sucrose diet for 4 weeks prior to mating, and through gestation day E8.5. In these pregnancies as well, numbers of lipid droplets and their sizes were significantly elevated compared to normal FVB pregnancies (Figure 3, Panels I–L). Taken together, our results revealed excessive lipid accumulation in visceral yolk sac under conditions of diabetic pregnancy, and upon HFD feeding.

### Expression of lipid transporter proteins in yolk sacs from pregnancies affected by maternal diabetes and obesity

**FIGURE 4:**
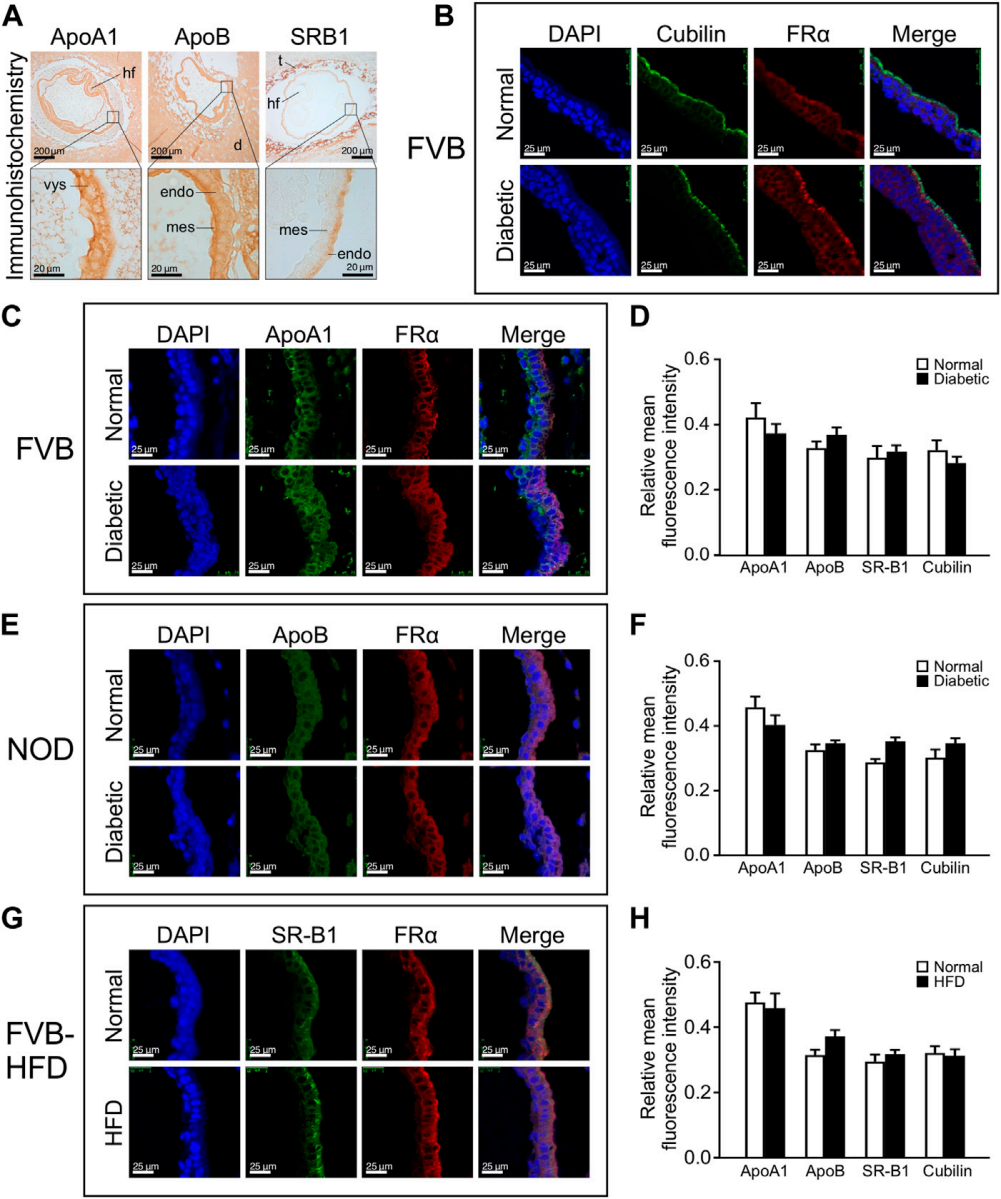
Lipid transporter protein expression in yolk sacs is unaffected by maternal metabolic disease or high fat diet-feeding. Expression of lipid transporter proteins was detected by immunohistochemistry and immunofluorescence on serial sections from paraffin-embedded whole decidua isolated at E8.5. The apical surface of the endodermal layer of the yolk sac is facing towards the right in all images. Panel **A:** Immunohistochemical staining for ApoA1, ApoB, and SR-B1 in specimen from a normal FVB pregnancy. Areas of magnification (lower images) are indicated by frames in the upper images. Abbreviations: hf = head fold of embryo; d = deciduum; t = trophoblast layer; vys = visceral yolk sac; mes = mesodermal layer of visceral yolk sac; endo = endodermal layer of visceral yolk sac. Panel **B**: Positive identification of the endodermal layer of the visceral yolk sac by immunofluorescence staining for Cubilin and Folate Receptor 1 (here detected with an antibody against FRα). Cubilin is very selectively located only on the apical surface of the visceral endoderm, whereas Folate Receptor 1 staining detects the entire endoderm layer and was therefore used in all immunofluorescence assays. Panel **C**: Immunofluorescence detection of ApoA1 expression in yolk sac from FVB pregnant mice. ApoA1 expression is present in endodermal, Folate Receptor 1-positive cells as well as FolR1-negative cells of mesodermal origin. Panel **D**: Quantification of fluorescence for each lipid transporter relative to the DAPI signal intensity in cells expressing the respective protein. No significant differences were found for lipid transporter expression between yolk sacs from diabetic compared to normoglycemic FVB dams. N = six to eight samples from 3 normoglycemic FVB pregnancies, n = seven to nine samples from 2 diabetic FVB pregnancies, sample numbers varied depending on protein investigated. Panel **E**: Immunofluorescence detection of ApoB in yolk sac from NOD pregnant mice. Panel **F**: Quantification of relative expression levels did not reveal statistically significant differences in yolk sac lipid transporter expression between diabetic and normoglycemic NOD pregnancies. N = 7–20 samples from 3 normoglycemic NOD pregnancies, n = 3–16 samples from 2 diabetic NOD pregnancies. Panel **G**: Immunofluorescence detection of SR-B1 expression in yolk sac from FVB dams where one group had been fed a high fat diet for 4 weeks prior to mating and until sacrifice. SR-B1 signal is predominantly located at the apical surface of the endodermal cells layer, coincident with enriched Folate Receptor 1 localization. An apparently stronger signal for SR-B1 in the HFD condition was only detected in this sample, and was not consistent after quantification of multiple specimen (see Panel H). N = 12–15 samples from chow-fed FVB pregnancies, n = 8–17 samples from high-fat-diet-fed FVB pregnancies. Panel **H**: Quantification of relative expression levels did not reveal statistically significant differences in yolk sac lipid transporter expression between high-fat-diet-fed and chow-diet-fed dams. Results were first averaged over each pregnancy and then the respective experimental group, presented as mean ± SD, and statistical significance at *p* < 0.05 would be indicated by asterisks if detected.

We then investigated whether lipid accumulation could be caused by aberrant expression of lipid transport proteins. We focused on ApoA1, ApoB and SR-B1, based upon prior reports of yolk sac expression, and the appearance of neural tube defects in mouse mutants for ApoB and SR-B1, which is a major transporter of cholesterol. The cellular localization of lipid transport proteins was revealed by immunohistochemistry staining for ApoA1, ApoB and SR-B1, on serial sections of entire paraffin-embedded decidua. Figure 4 Panel A depicts immunohistochemical staining for ApoA1 and ApoB, which are widely expressed in the decidua, as well as embryonic tissues and the yolk sac. Within the visceral yolk sac, ApoA1 was found expressed in endoderm as well as mesodermal cells (see magnification), whereas more intense ApoB signal was observed at the mesodermal yolk sac surface. SR-B1 was not broadly expressed in decidua, but exhibited prominent staining in trophoblasts; within the visceral yolk sac, a moderate signal was observed, which was specifically localized to the endodermal layer of the yolk sac (see magnification).

To identify positively the endodermal layer upon fluorescence staining, we used antibodies against Folate receptor 1, which we had previously shown to be exclusively expressed in visceral endoderm cells at this developmental stage (Salbaum et al., 2009). Figure 4 Panel B depicts Folate Receptor 1 staining in endodermal cells, in which expression of the Vitamin B12 receptor Cubilin is selectively localized only at the apical surface; we therefore continued to use Folate Receptor 1 to visualize the whole endodermal cell layer. While all three lipid and both vitamin transporters were examined in the three experimental models, Figure 4 Panels C, E, and G depict representative staining images for just one each of the lipid transporters in either of the experimental models, with quantification of all transporters in all the experimental models depicted in Panels D, F, and H. We did not observe altered cellular localization under conditions of exposure to maternal metabolic disease when compared to normal pregnancies, indicating that the cell-type specificity of expression was preserved for all examined nutrient transporters. Quantification was achieved by determining fluorescence intensities of lipid transporter staining, and for cells expressing the transporters, the intensities of DAPI staining were also determined, employing NIH ImageJ. Relative fluorescence intensities were calculated as the ratio of total fluorescence intensity of these lipid transporter proteins over DAPI intensity in those cells expressing the respective protein. No significant differences of expression levels were observed for ApoA1, ApoB, SR-B1 and Cubilin in the yolk sacs from normal, diabetic and HFD pregnancies (Figure 4 Panels D, F, and H, respectively). Taken together, these results indicate that metabolic status or high-fat-diet-feeding of the dam did not affect lipid transporter expression in the yolk sac at E8.5 days of gestation.

These results are consistent with expression at the RNA level, as represented in an RNAseq. dataset derived from yolk sacs of NOD diabetic and normoglycemic pregnancies at E8.5 (Table 1) that was queried for lipid transporters, and other genes involved in lipid droplet formation and processing. None of the 77 genes in this list exhibit statistically significant differences between metabolic conditions on the basis of adjusted *p*-values. When raw *p*-values were considered, only the first 9 genes (including Cideb) displayed significance, but their fold-changes were moderate at best, and expression levels low. Notably, among the highly expressed genes were ApoE, ApoA1, ApoA2, ApoA4, ApoB, ApoM, HDL binding protein, Cubilin and Megalin (Lrp2), Sortin nexins 1, 3, 5 and 6, and Microsomal triglyceride transfer protein. Yet, their expression did not significantly differ between metabolic conditions. When interrogated for all genes with Slc (solute carrier) nomenclature, this dataset also did not reveal significant differences for over 200 nutrient transporter genes expressed at this stage (Kappen et al., 2022). Taking together our quantitative and qualitative data, they do not provide evidence that altered gene expression in lipid-related pathways could be invoked as causes for the excess lipid accumulation in any of our three experimental models.

**TABLE 1:** Expression levels of genes with relevance to lipid transport and processing.

MGI acc. #	Symbol	Description	meanCtrl	meanTreat	FC	Padj
96,828	Lrp1	low density lipoprotein receptor-related protein 1	61.62	77.05	1.25	0.23
1,915,091	Atg3	autophagy related 3	185.08	160.34	-1.15	0.23
1,914,776	Atg12	autophagy related 12	260.70	226.11	-1.15	0.26
2,443,882	Snx30	sorting nexin family member 30	133.17	154.43	1.16	0.27
2,387,801	Snx17	sorting nexin 17	89.29	104.90	1.17	0.31
893,578	Scarb1	scavenger receptor class B, member 1	188.06	214.59	1.14	0.38
1,916,428	Snx5	sorting nexin 5	1388.80	1270.25	-1.09	0.39
1,277,186	Atg5	autophagy related 5	117.78	104.01	-1.13	0.39
1,270,844	Cideb	cell death-inducing DNA fragment. factor, α subunit-like effector B	86.79	69.85	-1.24	0.41
1,918,190	Dap	death-associated protein	231.23	249.87	1.08	0.43
2,137,642	Snx18	sorting nexin 18	144.30	157.18	1.09	0.44
1,923,159	Vmp1	vacuole membrane protein 1	479.03	418.94	-1.14	0.45
1,916,400	Snx4	sorting nexin 4	387.77	354.81	-1.09	0.45
96,765	Ldlr	low density lipoprotein receptor	88.45	109.61	1.24	0.47
1,931,027	Stx12	syntaxin 12	256.67	234.69	-1.09	0.47
1,919,433	Snx6	sorting nexin 6	631.00	569.02	-1.11	0.47
1,929,480	Lrp10	low-density lipoprotein receptor-related protein 10	107.38	120.58	1.12	0.48
1,933,830	Enpp5	ectonucleotide pyrophosphatase/phosphodiesterase 5	55.98	48.42	-1.16	0.48
1,891,421	Mesd	mesoderm development LRP chaperone	343.36	317.54	-1.08	0.48
1,860,508	Abcb10	ATP-binding cassette, sub-family B (MDR/TAP), member 10	170.29	153.53	-1.11	0.48
2,444,575	Soga1	suppressor of glucose, autophagy associated 1	48.05	58.00	1.21	0.49
1,915,054	Snx2	sorting nexin 2	333.99	298.15	-1.12	0.49
97,783	Psap	Prosaposin	121.00	147.65	1.22	0.49
1,919,331	Snx12	sorting nexin 12	399.97	362.69	-1.10	0.51
1,351,617	Abca3	ATP-binding cassette, sub-family A (ABC1), member 3	103.74	115.82	1.12	0.51
1,916,823	Hilpda	hypoxia inducible lipid droplet associated	179.63	201.11	1.12	0.57
1,891,828	Becn1	beclin 1, autophagy related	195.76	182.52	-1.07	0.57
1,347,061	Abcg2	ATP binding cassette subfamily G member 2 (Junior blood group)	566.04	527.80	-1.07	0.57
1,914,090	Wdr45b	WD repeat domain 45B	353.17	326.68	-1.08	0.58
2,140,175	Ldlrap1	low density lipoprotein receptor adaptor protein 1	125.57	144.10	1.15	0.58
1,923,811	Snx7	sorting nexin 7	54.20	47.67	-1.14	0.60
109,533	Abcb7	ATP-binding cassette, sub-family B (MDR/TAP), member 7	61.15	56.46	-1.08	0.61
1,196,458	Scarb2	scavenger receptor class B, member 2	49.56	53.44	1.08	0.62
88,051	Apoa4	apolipoprotein A-IV	614.66	683.86	1.11	0.63
1,921,968	Snx16	sorting nexin 16	126.22	116.38	-1.08	0.63
2,443,816	Snx8	sorting nexin 8	472.71	425.94	-1.11	0.64
2,138,856	Ldlrad3	low density lipoprotein receptor class A domain containing 3	46.09	52.74	1.14	0.64
88,055	Apoc3	apolipoprotein C-III	67.97	61.57	-1.10	0.65
1,349,216	Abcd3	ATP-binding cassette, sub-family D (ALD), member 3	405.09	382.79	-1.06	0.66
95,794	Lrp2	low density lipoprotein receptor-related protein 2	681.95	635.23	-1.07	0.66
1,351,658	Abcf1	ATP-binding cassette, sub-family F (GCN20), member 1	310.29	338.78	1.09	0.69
1,298,218	Lrp6	low density lipoprotein receptor-related protein 6	127.84	135.65	1.06	0.71
1,914,155	Plin3	perilipin 3	452.75	432.97	-1.05	0.71
1,928,395	Snx1	sorting nexin 1	604.79	562.58	-1.08	0.74
1,915,065	Sec14l2	SEC14-like lipid binding 2	336.70	316.78	-1.06	0.74
87,920	Plin2	perilipin 2	358.57	337.58	-1.06	0.77
1,915,367	Apool	apolipoprotein O-like	58.10	53.76	-1.08	0.77
88,050	Apoa2	apolipoprotein A-II	565.05	613.99	1.09	0.79
106,926	Mttp	microsomal triglyceride transfer protein	611.78	590.73	-1.04	0.79
1,913,865	Atg4b	autophagy related 4B, cysteine peptidase	111.02	106.61	-1.04	0.80
1,915,368	Atg101	autophagy related 101	130.47	124.81	-1.05	0.81
96,829	Lrpap1	low density lipoprotein receptor-related protein associated protein 1	380.07	362.50	-1.05	0.82
1,919,232	Snx10	sorting nexin 10	84.36	87.30	1.03	0.82
1,352,447	Abcc2	ATP-binding cassette, sub-family C (CFTR/MRP), member 2	133.90	142.19	1.06	0.83
1,195,458	Abce1	ATP-binding cassette, sub-family E (OABP), member 1	505.65	486.33	-1.04	0.83
99,256	Hdlbp	high density lipoprotein (HDL) binding protein	993.29	974.33	-1.02	0.84
96,820	Lpl	lipoprotein lipase	49.52	52.55	1.06	0.84
88,057	Apoe	apolipoprotein E	4485.10	4625.62	1.03	0.85
1,351,656	Abcf3	ATP-binding cassette, sub-family F (GCN20), member 3	47.84	49.52	1.04	0.85
1,927,471	Lsr	lipolysis stimulated lipoprotein receptor	480.94	497.06	1.03	0.85
2,155,664	Snx14	sorting nexin 14	71.85	69.39	-1.04	0.87
108,498	Nbr1	NBR1, autophagy cargo receptor	203.86	199.29	-1.02	0.87
1,931,256	Cubn	cubilin (intrinsic factor-cobalamin receptor)	772.08	744.74	-1.04	0.90
1,923,809	Atg2b	autophagy related 2B	77.40	80.25	1.04	0.90
1,914,421	Dram2	DNA-damage regulated autophagy modulator 2	71.97	69.26	-1.04	0.91
95,568	Folr1	folate receptor 1 (adult)	334.00	325.21	-1.03	0.92
88,053	Apoc1	apolipoprotein C-I	116.26	120.19	1.03	0.92
88,049	Apoa1	apolipoprotein A-I	1988.87	1938.81	-1.03	0.94
1,278,315	Lrp5	low density lipoprotein receptor-related protein 5	56.13	57.65	1.03	0.94
1,924,290	Atg16l1	autophagy related 16-like 1 (S. cerevisiae)	60.69	60.28	-1.01	0.96
1,351,644	Abcc5	ATP-binding cassette, sub-family C (CFTR/MRP), member 5	254.45	253.78	-1.00	0.96
88,052	Apob	apolipoprotein B	1775.25	1812.33	1.02	0.97
1,860,188	Snx3	sorting nexin 3	696.45	706.37	1.01	0.97
1,351,657	Abcf2	ATP-binding cassette, sub-family F (GCN20), member 2	85.40	87.06	1.02	0.97
2,443,111	Abcc4	ATP-binding cassette, sub-family C (CFTR/MRP), member 4	224.01	222.07	-1.01	0.98
1,923,992	Snx27	sorting nexin family member 27	354.27	353.60	-1.00	0.99
1,930,124	Apom	apolipoprotein M	1215.12	1213.62	-1.00	1.00

The GO annotations for an existing RNA seq. data set of E8.5 yolk sacs from normoglycemic and diabetic pregnancies of the NOD mouse strain were queried with search terms lipid, droplet, lipoprotein, lysosome, lipolysis, autophagy, Lrp, Snx, Perilipin, Cubilin, Enpp, and FolR1, and the extracted gene list was adjusted for expression level as described in the Methods section. The results in this table are sorted by adjusted *p*-value (from smallest to largest). The differences between controls and experimental samples were not significant.

### Decreased colocalization of lipid droplets and lysosomes in yolk sacs of diabetic pregnancies and with high-fat diet feeding

**FIGURE 5:**
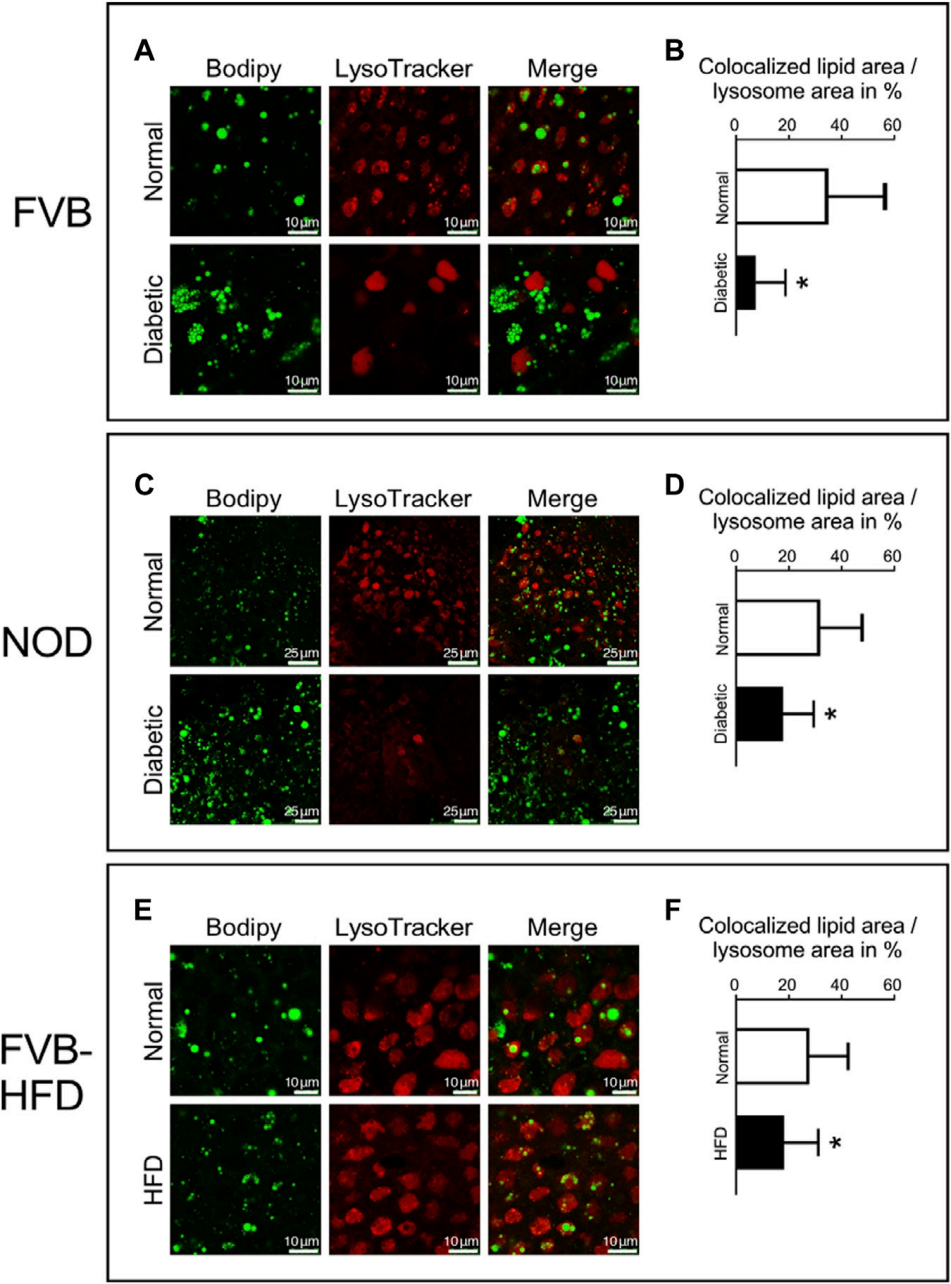
Decreased colocalization of lipid droplets and lysosomes in yolk sacs from diabetic and obese pregnancies. Live yolk sac tissue was stained with LysoTracker dye and Bodipy. Panel **A**: Yolk sac from normoglycemic and diabetic FVB pregnancies; bigger lipid droplets were observed in the lumen of lysosomes in the normal yolk sac. Panel **B**: The areas depicting lipid droplets colocalized with lysosomes were identified and quantified, and the areas of the corresponding individual lysosomes were measured. The ratios of the areas occupied by colocalized lipid droplets over the corresponding individual lysosome areas were calculated (depicted in %). Lipid droplet colocalization with lysosomes was significantly decreased in yolk sacs from diabetic FVB dams. Sample size: N = 18 yolk sacs from 5 normoglycemic FVB dam pregnancies, n = 16 yolk sacs from 6 diabetic FVB dam pregnancies. Panel **C**: Yolk sacs from normoglycemic and diabetic NOD pregnancies were analyzed for lipid droplet colocalization with lysosomes (smaller magnification than Panel A). Panel **D**: Quantification reveals that lipid droplet colocalization with lysosomes was significantly decreased in yolk sacs from diabetic NOD dams (n = 18 yolk sacs from 6 diabetic NOD pregnancies) when compared to normoglycemic NOD pregnancies (n = 21 yolk sacs from 5 pregnancies). Panel **E**: Lipid droplet colocalization with lysosomes analyzed in yolk sacs from chow- and high-fat-diet-fed FVB pregnant dams. Panel **F**: Lipid droplet colocalization with lysosomes was significantly decreased in yolk sacs from HFD-fed pregnant FVB dams (n = 14 yolk sacs from 4 pregnancies) when compared to yolk sacs from chow-fed FVB pregnancies (n = 18 yolk sacs from 5 pregnancies). Data presented as mean ± SD, first averaged over each pregnancy, then for each respective experimental group; asterisks indicate *p* < 0.05, when comparing experimental and control groups using two-tailed t-tests.

We then considered the possibility that lipid processing could be altered in yolk sacs with excessive lipid accumulation. Lipid mobilization from droplets involves the autophagosome and lysosomes (Brent et al., 1990; Zohn and Sarkar, 2010). To investigate lipid processing in lysosomes, fresh visceral yolk sacs were isolated at E8.5 days of gestation from normal, diabetic and HFD pregnancies. Lysosomes were stained with LysoTracker dye, and lipids with Bodipy, respectively. Figure 5 Panel A shows that in normoglycemic pregnancy, many lipid droplets are colocalized with the lumen of lysosomes in visceral yolk sac cells. In yolk sac from FVB-diabetic pregnancies, however, more lipid droplets are present, often clustering together, and fewer of them are associated with lysosomes. A corresponding situation is detected in NOD diabetic pregnancies (Figure 5 Panel C). The areas of colocalization, and the corresponding individual lysosome areas were identified and quantified, by using the NIH ImageJ plugin Colocalization Image Creator, and then we calculated the ratio of colocalized lipids relative to the corresponding individual lysosome area size. The fraction of colocalization was significantly decreased in yolk sacs from FVB-diabetic pregnancies compared to normal pregnancies (Figure 5 Panel B). Likewise, there was significantly decreased colocalization of lipid droplets with lysosomes in yolk sacs from NOD-diabetic pregnancies (Figure 5 Panel D). These results are consistent with a lower rate of incorporation of lipid droplets into lysosomes, suggesting that lipid accumulation could be due to abnormal lipid processing in yolk sac cells.

Notably, in FVB diabetic pregnancies, the lysosomes appeared to be larger, and more uniformly stained when compared to normal yolk sac (Figure 5 Panel A, lower middle). The number of lysosomes also seemed to be reduced in NOD diabetes-exposed yolk sacs (Figure 5 Panel C lower middle), although we were unable to quantify these observations due to the lack of a reference for normalization to cell size or number when staining live tissue. In contrast, in yolk sac from dams fed high-fat diet, the size and number of lysosomes, as well as their staining appearance is indistinguishable from control yolk sac cells (Figure 5 Panel E, middle images), with appreciable but -albeit reduced-colocalization of lipid droplets compared to control (Figure 5, Panel F). Thus, even though appreciable numbers of lysosomes are present, they contain fewer lipid droplets. Taken together, our results are indicative of a reduced rate of lipid processing in yolk sacs with excess lipid accumulation, which conceivably would be exacerbated by a lower number of lysosomes in yolk sacs from diabetic pregnancies.

## Discussion

Our study reveals excessive lipid accumulation in pregnancies affected by both maternal diabetes and obesity, using *in vivo*-derived visceral yolk sacs isolated at gestational day E8.5. A particular strength of our approach were the large sample numbers, which allowed us to balance the biological variability inherent in mouse pregnancies. Furthermore, in addition to microscopic identification of structures and cells, we quantified lipid droplets based upon volumetric rendering from consecutive (Z-stack) confocal images. These data revealed both increased quantity and larger sizes of lipid droplets in yolk sac cells that were exposed to maternal metabolic disease. The use of two different mouse strains and of two independent models of diabetic pregnancy make our findings particularly compelling. We acknowledge as weaknesses of the present study the lack of functional measurements of nutrient transport, as well as the limited information on relevance of lipid accumulations for cellular metabolism in the yolk sac itself, and on consequences for nutrient metabolism in the embryo.

Our results differ from a previous report that observed fewer lipid droplets in yolk sacs from E10 rat conceptuses that were cultured *in vitro* for 2 days in medium containing high levels of glucose (Reece et al., 1994). This was associated with release of oleic acid into the medium, interpreted by the authors as evidence for potential lipid deficiency in the embryo (Pinter et al., 1988). Subsequently, it was suggested that the yolk sac in diabetic pregnancy may also be deficient in essential fatty acids (Pinter et al., 1986a; Reece et al., 1994). In contrast, our work demonstrates excessive lipid accumulation in yolk sac freshly isolated from diabetic pregnancies, albeit at earlier stages than investigated previously. Thus, lipids are either taken up from the maternal circulation, where they are in excess supply due to maternal hyperlipidemia in diabetic pregnancies, or they would have to be synthesized in visceral yolk sac from the excess msternal glucose under hyperglycemic conditions. We have previously shown that in addition to Glut3, the visceral yolk sac expresses the high-capacity glucose transporter Glut2, specifically in the endodermal cell layer (Kappen et al., 2022), lending plausibility to excessive glucose uptake. We here show that lipid transporters ApoA1, ApoB, SR-B1 and Cubilin are also expressed in the visceral yolk sac, with Cubilin and SR-B1 restricted to the endodermal layer, while ApoA1 and ApoB are also found in the mesodermal compartment of the visceral yolk sac, consistent with prior literature (Shi and Heath, 1984; Farese et al., 1996; Terasawa et al., 1999; Strope et al., 2004; Santander et al., 2013). Maternal diabetes did not affect expression of these lipid transporters, but this does not exclude the possibility that they could be importing lipids or fatty acids at a higher rate under conditions of hyperlipidemia. When dams had normal blood glucose levels but were fed a high-fat content diet, we also observed excess lipid accumulation in yolk sacs from these pregnancies. Our finding that excess lipid accumulation can occur with normoglycemia suggests that excess lipid supply might be associated with excess lipid supply, either through diet or maternal hyperlipidemia, with glucose playing only a minor role.

Taken together, these results argue against an endocytic deficit in the yolk sac, as was previously postulated for diabetic pregnancies, based upon reduced Horseradish peroxidase uptake (Reece et al., 1989) by yolk sac in rat conceptuses cultured *in vitro* in high concentrations of glucose. For mouse conceptuses at early somite stages, amino acid and protein uptake into the visceral yolk sac was found reduced only after prolonged exposure to high concentrations of glucose (Hunter and Sadler, 1992). Thus, we cannot rule out that the excess accumulation of lipids at the stages we investigated here could potentially lead to decreased nutrient yolk sac uptake at later stages; additional gestational time points would have to be investigated to address this possibility.

Lipids taken up by or synthesized by the yolk sac, or present in lipid droplets, require processing before they can be transported to the embryo (Zohn and Sarkar, 2010). Autophagy is required for lipid droplet turnover in yolk sac cells, as evidenced by increased lipid accumulation in yolk sacs of embryos lacking the essential autophagy gene, Atg7 (Singh et al., 2009). Lipophagy is a selective form of autophagy particularly of intracellular lipid droplets. The process of lipophagy begins with formation of double membraned autophagosomes that engulf the lipid droplets, and then merge with lysosomes to form the autolysosome for further degradation (Singh, 2010). For other cell types, it was shown that excessive size of lipid droplets inhibits uptake into the autophagosome (Singh et al., 2009) and prevents lipophagy (Schott et al., 2019). Thus, the larger lipid droplets accumulated in our models may also prevent proper lipid processing under conditions of maternal metabolic disease. Defective autophagy and lipophagy therefore result in excessive lipid accumulation (Singh, 2010), and excessive lipid loads -in a negative feedback-loop- then further inhibit lipophagy (Knaevelsrud and Simonsen, 2012). Our results, namely that fewer lipid droplets colocalize with lysosomes, are suggestive of similar defects in lysosomal lipid processing in yolk sac cells in conditions of maternal diabetes or obesity.

It is currently unknown to what extent the increased lipid accumulation is detrimental to the yolk sac endoderm cells themselves. As lipid droplets are enveloped by perilipins (Sztalryd and Brasaemle, 2017) and other lipid-droplet-associated proteins, yolk sac cells undergoing excess lipid accumulation most likely have to divert considerable resources to the synthesis of these proteins. Then, it is conceivable that yolk sac cells with excessive lipid accumulation -owing to defective lipid processing- experience an energy deficit, which may affect their capacity to transport nutrients to the embryo. Intriguingly, reduction of rough endoplasmic reticulum, ribosomes and mitochondria number have been observed in yolk sac cells of rat conceptuses cultured in high glucose (Pinter et al., 1986a), consistent with biosynthetic insufficiency. Additionally, the mesodermal compartment of the yolk sac is affected in diabetic pregnancies at later stages, in that vascularization was reported to be reduced in diabetic pregnancies of mice (Pinter et al., 1999; Pinter et al., 2004) and rats (Zabihi et al., 2007).

Lipids are required for membrane and organelle synthesis in normal embryo development, and in yolk sac cells. Nascent VLDL was found by ultrastructural analysis within the luminal spaces of the rough endoplasmic reticulum and Golgi apparatus in yolk sac visceral endoderm cells at E7.5 and E8.5, suggesting a vital role of yolk sac in providing lipids and lipid-soluble nutrients to embryos during the early phases of mouse development (Terasawa et al., 1999). Transport of lipids out of yolk sac cells involves ApoB and the microsomal triglyceride transfer protein (MTP), encoded by the mouse Mttp gene. MTP is required for assembly of ApoB-containing lipoprotein complexes, and Mttp-knockout mice exhibit embryonic lethality (Raabe et al., 1998), with marked lipid accumulation at the basolateral surfaces of yolk sac visceral endodermal cells at E9.5. Homozygous mutant embryos displayed retarded growth, and failure of anterior neural tube closure by E10.5, as well as exencephaly at E14.5. (Raabe et al., 1998). Likewise, ApoB is critical to lipoprotein assembly in visceral yolk sac endodermal cells (Farese et al., 1996), and deficiency of ApoB is associated with failure to close the neural tube, exencephaly, hydrocephaly, embryonic lethality, and embryo apoptosis and resorption, with the spectrum of phenotypes depending on the particular mouse model (Homanics et al., 1993; Farese et al., 1995; Huang et al., 1995). In the ApoB mutants, excessive lipid droplet accumulation in yolk sac endodermal cells was accompanied by deficiencies of Vitamin E and of cholesterol in the embryo (Farese et al., 1996), and embryonic defects could be ameliorated by alpha-tocopherol supplementation (Homanics et al., 1993; Farese et al., 1996). Mouse embryos deficient for the HDL transporter SR-B1 experience Vitamin E-deficiency, and are prone to develop neural tube defects (Santander et al., 2013; Santander et al., 2017). Shortage of maternal cholesterol also has been shown to result in multiple congenital birth defect in the offspring, including holoprosencephaly, neural tube defects, and heart and limb anomalies (Cooper et al., 1998). Based upon these findings, we expect that embryos whose yolk sacs undergo excessive lipid accumulation in diabetic pregnancies would also exhibit deficiencies of cholesterol and Vitamin E. The benefit of Vitamin E supplementation in reducing the incidence of developmental defects in such pregnancies (Sivan et al., 1996) provides strong support for this notion; cholesterol supplementation has not been reported in diabetic pregnancies to date.

In previous studies, supplementations of Arachidonic acid and its derivative prostaglandin E_2_ (PGE_2_) were shown to reduce the incidence of embryonic malformation caused by maternal diabetes in mouse and rat embryos *in vivo*, and in *vitro* cultures under high glucose high oxygen conditions (Goldman et al., 1985; Pinter et al., 1988; Baker et al., 1990; Goto et al., 1992). Lower levels of prostaglandin E2 were detected in rat embryos from diabetic pregnancies (Wentzel and Eriksson, 2005), and in rat embryos cultured in high glucose (Wentzel and Eriksson, 2005). Embryos contained less Arachidonic acid within the phospholipid fraction when conceptuses were cultured in high glucose (Pinter et al., 1988), but Arachidonic acid was increased in the yolk sacs under these conditions, indicating that it may also be captured in the accumulating lipid droplets, and possibly unavailable as a precursor for PGE_2_ production. In humans, significant reduction in yolk sac levels of PGE_2_ in early pregnancy of diabetic women has been reported (Schoenfeld et al., 1995). Consistent with this, single lipid species supplementation has been shown to reduce embryonic malformations (Reece et al., 1996; Wiznitzer et al., 1999; Wentzel and Eriksson, 2005; Reece et al., 2006) in animal models. Taking our results together with prior evidence, it appears that multiple lipid species could be of insufficient availability for optimal growth of embryos in diabetic pregnancies.

While our result indicate that lipid processing is defective in yolk sac endodermal cells under diabetic and obesity conditions, it remains to be determined to which extent the processing of other nutrients, such as proteins or glucose for examples, is also altered. For examples, in yolk sacs from mice with a targeted disruption of the gene encoding ADP-ribosylation factor-like 8B, the trafficking of lysosomes is defective, leading to accumulation of proteins in late endocytic organelles and reduced availability of free amino acids to the developing embryo (Oka et al., 2017). Impairment of transport function has also been described for yolk sac deficient in Pax-Interacting Protein 1-associated glutamate rich protein 1a (Pagr1a), due to an absence of apical vacuoles (Kumar et al., 2014). An excess number of apical vacuoles is also associated with defects in lysosomal processing in the yolk sac of mutants with ablation of both Sorting nexins 1 and 2 (Snx1, Snx2 double mutants), resulting in developmental delay and neural tube defects in the mutant embryos (Schwarz et al., 2002). Moreover, Autotaxin, an ectonucleotide pyrophosphatase/phosphodiesterase, encoded by the Enpp2 gene, is required for conversion of smaller lysosomes to larger size (Koike et al., 2009), which is essential for proper nutrient processing in yolk sac endodermal cells. Enpp2 germ-line mutant embryos suffer growth retardation, failure of turning and embryonic lethality (Koike et al., 2010), and embryos with disruption of Enpp2 in the neural tube (*via* Sox1-driven cre-mediated recombination) exhibit neural tube defects (van Meeteren et al., 2006). Taken together, these evidences underscore the importance of effective lysosome processing for supply of nutrients to the developing embryo. Furthermore, insofar the multiple mouse mutant models discussed here have demonstrated causal links between defective yolk sac lipid metabolism and neural tube closure, they provide support for our conclusion that defective lipid processing in yolk sac cells possibly underlies the risk for neural tube defects in pregnancies affected by maternal diabetes, and by inference, obesity.

Previous studies have observed developmental delay and growth retardation of the embryos from diabetic mice and rats (Eriksson et al., 1984; Zhao et al., 2017), which would be consistent with generalized nutrient deficit. If a wide variety of essential nutrients is in limited supply, this -conceivably- could be the cause of embryonic growth restrictions, and the reduced growth of placenta in diabetic pregnancies that we reported previously (Kappen et al., 2011; Salbaum et al., 2011). Interestingly, growth restriction in placenta and embryos was more pronounced when the diabetic pregnant dams were fed a diet with higher lipid content (Kappen et al., 2011, 2012), and associated with a higher rate of neural tube defects (Kappen et al., 2011). It remains to be determined whether the extent of lipid accumulation is even more excessive under these dietary conditions, and whether the very high-fat-diet we showed here to be sufficient for excessive lipid droplet accumulation in yolk sac in normoglycemic FVB dams would exacerbate the lysosomal dysfunction in diabetic pregnancies.

Finally, it is intriguing to speculate whether compounds can be identified that either stimulate lipid release from droplets, or promote lipid processing in the yolk sac, and as a consequence, might enhance lipid transport to the embryo. The expectation would be that restored supply of vital nutrients to embryos in diabetic pregnancies should also lower the incidence of neural tube defects. Considering that neural tube closure proceeds within a relatively short time frame, even short-term treatment might be effective if given just before the neural tube closure process commences.

## Data Availability

The datasets presented in this study can be found in online repositories. The names of the repository/repositories and accession number(s) can be found in the article/supplementary material.

## References

[B1] Aoyama M. Sun-Wada G. H. Yamamoto A. Yamamoto M. Hamada H. Wada Y. (2012). Spatial restriction of bone morphogenetic protein signaling in mouse gastrula through the mVam2-dependent endocytic pathway. Dev. Cell 22 (6), 1163–1175. 10.1016/j.devcel.2012.05.009 22698281

[B2] Atkinson M. A. Leiter E. H. (1999). The NOD mouse model of type 1 diabetes: As good as it gets? Nat. Med. 5 (6), 601–604. 10.1038/9442 10371488

[B3] Baker L. Piddington R. Goldman A. Egler J. Moehring J. (1990). Myo-inositol and prostaglandins reverse the glucose inhibition of neural tube fusion in cultured mouse embryos. Diabetologia 33 (10), 593–596. 10.1007/BF00400202 2257996

[B4] Brent R. L. Beckman D. A. Jensen M. Koszalka T. R. (1990). Experimental yolk sac dysfunction as a model for studying nutritional disturbances in the embryo during early organogenesis. Teratology 41 (4), 405–413. 10.1002/tera.1420410406 2187260

[B5] Cooper M. K. Porter J. A. Young K. E. Beachy P. A. (1998). Teratogen-mediated inhibition of target tissue response to Shh signaling. Science 280 (5369), 1603–1607. 10.1126/science.280.5369.1603 9616123

[B6] Eriksson U. J. Lewis N. J. Freinkel N. (1984). Growth retardation during early organogenesis in embryos of experimentally diabetic rats. Diabetes 33 (3), 281–284. 10.2337/diab.33.3.281 6698318

[B7] Farese R. V. Jr. Cases S. Ruland S. L. Kayden H. J. Wong J. S. Young S. G. (1996). A novel function for apolipoprotein B: Lipoprotein synthesis in the yolk sac is critical for maternal-fetal lipid transport in mice. J. Lipid Res. 37 (2), 347–360. 10.1016/s0022-2275(20)37621-5 9026532

[B8] Farese R. V. Jr. Ruland S. L. Flynn L. M. Stokowski R. P. Young S. G. (1995). Knockout of the mouse apolipoprotein B gene results in embryonic lethality in homozygotes and protection against diet-induced hypercholesterolemia in heterozygotes. Proc. Natl. Acad. Sci. U. S. A. 92 (5), 1774–1778. 10.1073/pnas.92.5.1774 7878058 PMC42602

[B9] Goldman A. S. Baker L. Piddington R. Marx B. Herold R. Egler J. (1985). Hyperglycemia-induced teratogenesis is mediated by a functional deficiency of arachidonic acid. Proc. Natl. Acad. Sci. U. S. A. 82 (23), 8227–8231. 10.1073/pnas.82.23.8227 3934670 PMC391476

[B10] Goto M. P. Goldman A. S. Uhing M. R. (1992). PGE2 prevents anomalies induced by hyperglycemia or diabetic serum in mouse embryos. Diabetes 41 (12), 1644–1650. 10.2337/diab.41.12.1644 1446806

[B11] Helle E. Priest J. R. (2020). Maternal obesity and diabetes mellitus as risk factors for congenital heart disease in the offspring. J. Am. Heart Assoc. 9 (8), e011541. 10.1161/JAHA.119.011541 32308111 PMC7428516

[B12] Homanics G. E. Smith T. J. Zhang S. H. Lee D. Young S. G. Maeda N. (1993). Targeted modification of the apolipoprotein B gene results in hypobetalipoproteinemia and developmental abnormalities in mice. Proc. Natl. Acad. Sci. U. S. A. 90 (6), 2389–2393. 10.1073/pnas.90.6.2389 8460149 PMC46092

[B13] Huang L. S. Voyiaziakis E. Markenson D. F. Sokol K. A. Hayek T. Breslow J. L. (1995). Apo B gene knockout in mice results in embryonic lethality in homozygotes and neural tube defects, male infertility, and reduced HDL cholesterol ester and apo A-I transport rates in heterozygotes. J. Clin. Invest. 96 (5), 2152–2161. 10.1172/JCI118269 7593600 PMC185864

[B14] Hunter E. S. 3rd Sadler T. W. (1992). The role of the visceral yolk sac in hyperglycemia-induced embryopathies in mouse embryos *in vitro* . Teratology 45 (2), 195–203. 10.1002/tera.1420450213 1615429

[B15] Kappen C. Kruger C. Jones S. Salbaum J. M. (2022). Nutrient transporter gene expression in the early conceptus-implications from two mouse models of diabetic pregnancy. Front. Cell Dev. Biol. 10, 777844. 10.3389/fcell.2022.777844 35478964 PMC9035823

[B16] Kappen C. Kruger C. Macgowan J. Salbaum J. M. (2012). Maternal diet modulates placenta growth and gene expression in a mouse model of diabetic pregnancy. PLoS One 7 (6), e38445. 10.1371/journal.pone.0038445 22701643 PMC3372526

[B17] Kappen C. Kruger C. MacGowan J. Salbaum J. M. (2011). Maternal diet modulates the risk for neural tube defects in a mouse model of diabetic pregnancy. Reprod. Toxicol. 31 (1), 41–49. 10.1016/j.reprotox.2010.09.002 20868740 PMC3035722

[B18] Knaevelsrud H. Simonsen A. (2012). Lipids in autophagy: Constituents, signaling molecules and cargo with relevance to disease. Biochim. Biophys. Acta 1821 (8), 1133–1145. 10.1016/j.bbalip.2012.01.001 22269166

[B19] Koike S. Keino-Masu K. Masu M. (2010). Deficiency of autotaxin/lysophospholipase D results in head cavity formation in mouse embryos through the LPA receptor-Rho-ROCK pathway. Biochem. Biophys. Res. Commun. 400 (1), 66–71. 10.1016/j.bbrc.2010.08.008 20692235

[B20] Koike S. Keino-Masu K. Ohto T. Sugiyama F. Takahashi S. Masu M. (2009). Autotaxin/lysophospholipase D-mediated lysophosphatidic acid signaling is required to form distinctive large lysosomes in the visceral endoderm cells of the mouse yolk sac. J. Biol. Chem. 284 (48), 33561–33570. 10.1074/jbc.M109.012716 19808661 PMC2785199

[B21] Kumar A. Lualdi M. Loncarek J. Cho Y. W. Lee J. E. Ge K. (2014). Loss of function of mouse Pax-Interacting Protein 1-associated glutamate rich protein 1a (Pagr1a) leads to reduced Bmp2 expression and defects in chorion and amnion development. Dev. Dyn. 243 (7), 937–947. 10.1002/dvdy.24125 24633704 PMC4153430

[B22] Leiter E. H. (2005). Nonobese diabetic mice and the genetics of diabetes susceptibility. Curr. Diab Rep. 5 (2), 141–148. 10.1007/s11892-005-0042-z 15794919

[B23] Leiter E. H. (1989). The genetics of diabetes susceptibility in mice. FASEB J. 3 (11), 2231–2241. 10.1096/fasebj.3.11.2673897 2673897

[B24] Martinez-Frias M. L. (1994). Epidemiological analysis of outcomes of pregnancy in diabetic mothers: Identification of the most characteristic and most frequent congenital anomalies. Am. J. Med. Genet. 51 (2), 108–113. 10.1002/ajmg.1320510206 8092185

[B25] Martinez-Frias M. L. Frias J. P. Bermejo E. Rodriguez-Pinilla E. Prieto L. Frias J. L. (2005). Pre-gestational maternal body mass index predicts an increased risk of congenital malformations in infants of mothers with gestational diabetes. Diabet. Med. 22 (6), 775–781. 10.1111/j.1464-5491.2005.01492.x 15910631

[B26] Mills J. L. (1982). Malformations in infants of diabetic mothers. Teratology 25 (3), 385–394. 10.1002/tera.1420250316 7051398

[B27] Nath A. K. Enciso J. Kuniyasu M. Hao X. Y. Madri J. A. Pinter E. (2004). Nitric oxide modulates murine yolk sac vasculogenesis and rescues glucose induced vasculopathy. Development 131 (10), 2485–2496. 10.1242/dev.01131 15128676

[B28] Oka M. Hashimoto K. Yamaguchi Y. Saitoh S. I. Sugiura Y. Motoi Y. (2017). Arl8b is required for lysosomal degradation of maternal proteins in the visceral yolk sac endoderm of mouse embryos. J. Cell Sci. 130 (20), 3568–3577. 10.1242/jcs.200519 28827407

[B29] Pani L. Horal M. Loeken M. R. (2002). Polymorphic susceptibility to the molecular causes of neural tube defects during diabetic embryopathy. Diabetes 51 (9), 2871–2874. 10.2337/diabetes.51.9.2871 12196484

[B30] Pavlinkova G. Salbaum J. M. Kappen C. (2009). Maternal diabetes alters transcriptional programs in the developing embryo. BMC Genomics 10 (1), 274. 10.1186/1471-2164-10-274 19538749 PMC2715936

[B31] Pinter E. Haigh J. Nagy A. Madri J. A. (2004). Hyperglycemia-induced vasculopathy in the murine conceptus is mediated via reductions of VEGF-A expression and VEGF receptor activation. Am. J. Pathol. 158 (4), 1199–1206. 10.1016/S0002-9440(10)64069-2 PMC1891927 11290536

[B32] Pinter E. Mahooti S. Wang Y. Imhof B. A. Madri J. A. (1999). Hyperglycemia-induced vasculopathy in the murine vitelline vasculature: Correlation with PECAM-1/CD31 tyrosine phosphorylation state. Am. J. Pathol. 154 (5), 1367–1379. 10.1016/S0002-9440(10)65391-6 10329590 PMC1866605

[B33] Pinter E. Reece E. A. Leranth C. Z. Garcia-Segura M. Hobbins J. C. Mahoney M. J. (1986a). Arachidonic acid prevents hyperglycemia-associated yolk sac damage and embryopathy. Am. J. Obstet. Gynecol. 155 (4), 691–702. 10.1016/s0002-9378(86)80001-1 3094372

[B34] Pinter E. Reece E. A. Leranth C. Z. Sanyal M. K. Hobbins J. C. Mahoney M. J. (1986b). Yolk sac failure in embryopathy due to hyperglycemia: Ultrastructural analysis of yolk sac differentiation associated with embryopathy in rat conceptuses under hyperglycemic conditions. Teratology 33 (1), 73–84. 10.1002/tera.1420330110 3738811

[B35] Pinter E. Reece E. A. Ogburn P. L. Jr. Turner S. Hobbins J. C. Mahoney M. J. (1988). Fatty acid content of yolk sac and embryo in hyperglycemia-induced embryopathy and effect of arachidonic acid supplementation. Am. J. Obstet. Gynecol. 159 (6), 1484–1490. 10.1016/0002-9378(88)90579-0 3144918

[B36] Qi Z. Fujita H. Jin J. Davis L. S. Wang Y. Fogo A. B. (2005). Characterization of susceptibility of inbred mouse strains to diabetic nephropathy. Diabetes 54 (9), 2628–2637. 10.2337/diabetes.54.9.2628 16123351

[B37] Raabe M. Flynn L. M. Zlot C. H. Wong J. S. Veniant M. M. Hamilton R. L. (1998). Knockout of the abetalipoproteinemia gene in mice: Reduced lipoprotein secretion in heterozygotes and embryonic lethality in homozygotes. Proc. Natl. Acad. Sci. U. S. A. 95 (15), 8686–8691. 10.1073/pnas.95.15.8686 9671739 PMC21137

[B38] Reece E. A. Pinter E. Homko C. Wu Y. K. Naftolin F. (1994). Review article: The yolk sac theory. J. Soc. Gynecol. Investig. 1 (1), 3–13. 10.1177/107155769400100103 9419739

[B39] Reece E. A. Pinter E. Leranth C. Hobbins J. C. Mahoney M. J. Naftolin F. (1989). Yolk sac failure in embryopathy due to hyperglycemia: Horseradish peroxidase uptake in the assessment of yolk sac function. Obstet. Gynecol. 74 (5), 755–762. 2812653

[B40] Reece E. A. Wu Y. K. Wiznitzer A. Homko C. Yao J. Borenstein M. (1996). Dietary polyunsaturated fatty acid prevents malformations in offspring of diabetic rats. Am. J. Obstet. Gynecol. 175 (4), 818–823. 10.1016/s0002-9378(96)80005-6 8885728

[B41] Reece E. A. Wu Y. K. Zhao Z. Dhanasekaran D. (2006). Dietary vitamin and lipid therapy rescues aberrant signaling and apoptosis and prevents hyperglycemia-induced diabetic embryopathy in rats. Am. J. Obstet. Gynecol. 194 (2), 580–585. 10.1016/j.ajog.2005.08.052 16458664

[B42] Ryan H. E. Lo J. Johnson R. S. (1998). HIF-1 alpha is required for solid tumor formation and embryonic vascularization. EMBO J. 17 (11), 3005–3015. 10.1093/emboj/17.11.3005 9606183 PMC1170640

[B43] Salbaum J. M. Finnell R. H. Kappen C. (2009). Regulation of folate receptor 1 gene expression in the visceral endoderm. Birth Defects Res. A Clin. Mol. Teratol. 85 (4), 303–313. 10.1002/bdra.20537 19180647 PMC2731486

[B44] Salbaum J. M. Kappen C. (2012). Responses of the embryonic epigenome to maternal diabetes. Birth Defects Res. A Clin. Mol. Teratol. 94 (10), 770–781. 10.1002/bdra.23035 22786762 PMC3882068

[B45] Salbaum J. M. Kruger C. Zhang X. Delahaye N. A. Pavlinkova G. Burk D. H. (2011). Altered gene expression and spongiotrophoblast differentiation in placenta from a mouse model of diabetes in pregnancy. Diabetologia 54 (7), 1909–1920. 10.1007/s00125-011-2132-6 21491160 PMC3882064

[B46] Santander N. G. Contreras-Duarte S. Awad M. F. Lizama C. Passalacqua I. Rigotti A. (2013). Developmental abnormalities in mouse embryos lacking the HDL receptor SR-BI. Hum. Mol. Genet. 22 (6), 1086–1096. 10.1093/hmg/dds510 23221804

[B47] Santander N. Lizama C. Parga M. J. Quiroz A. Perez D. Echeverria G. (2017). Deficient vitamin E uptake during development impairs neural tube closure in mice lacking lipoprotein receptor SR-BI. Sci. Rep. 7 (1), 5182. 10.1038/s41598-017-05422-w 28701710 PMC5507922

[B48] Schoenfeld A. Erman A. Warchaizer S. Ovadia J. Bonner G. Hod M. (1995). Yolk sac concentration of prostaglandin E2 in diabetic pregnancy: Further clues to the etiology of diabetic embryopathy. Prostaglandins 50 (3), 121–126. 10.1016/0090-6980(95)00084-4 8750208

[B49] Schott M. B. Weller S. G. Schulze R. J. Krueger E. W. Drizyte-Miller K. Casey C. A. (2019). Lipid droplet size directs lipolysis and lipophagy catabolism in hepatocytes. J. Cell Biol. 218 (10), 3320–3335. 10.1083/jcb.201803153 31391210 PMC6781454

[B50] Schwarz D. G. Griffin C. T. Schneider E. A. Yee D. Magnuson T. (2002). Genetic analysis of sorting nexins 1 and 2 reveals a redundant and essential function in mice. Mol. Biol. Cell 13 (10), 3588–3600. 10.1091/mbc.e02-03-0145 12388759 PMC129968

[B51] Shi W. K. Heath J. K. (1984). Apolipoprotein expression by murine visceral yolk sac endoderm. J. Embryol. Exp. Morphol. 81, 143–152. 10.1242/dev.81.1.143 6381629

[B52] Singh R. (2010). Autophagy and regulation of lipid metabolism. Results Probl. Cell Differ. 52, 35–46. 10.1007/978-3-642-14426-4_4 20865370 PMC4052896

[B53] Singh R. Kaushik S. Wang Y. Xiang Y. Novak I. Komatsu M. (2009). Autophagy regulates lipid metabolism. Nature 458 (7242), 1131–1135. 10.1038/nature07976 19339967 PMC2676208

[B54] Sivan E. Reece E. A. Wu Y. K. Homko C. J. Polansky M. Borenstein M. (1996). Dietary vitamin E prophylaxis and diabetic embryopathy: Morphologic and biochemical analysis. Am. J. Obstet. Gynecol. 175 (4), 793–799. 10.1016/s0002-9378(96)80001-9 8885724

[B55] Strope S. Rivi R. Metzger T. Manova K. Lacy E. (2004). Mouse amnionless, which is required for primitive streak assembly, mediates cell-surface localization and endocytic function of cubilin on visceral endoderm and kidney proximal tubules. Development 131 (19), 4787–4795. 10.1242/dev.01341 15342463

[B56] Sztalryd C. Brasaemle D. L. (2017). The perilipin family of lipid droplet proteins: Gatekeepers of intracellular lipolysis. Biochim. Biophys. Acta Mol. Cell Biol. Lipids 1862 (10), 1221–1232. 10.1016/j.bbalip.2017.07.009 28754637 PMC5595658

[B57] Terasawa Y. Cases S. J. Wong J. S. Jamil H. Jothi S. Traber M. G. (1999). Apolipoprotein B-related gene expression and ultrastructural characteristics of lipoprotein secretion in mouse yolk sac during embryonic development. J. Lipid Res. 40 (11), 1967–1977. 10.1016/s0022-2275(20)32420-2 10553000

[B58] van Meeteren L. A. Ruurs P. Stortelers C. Bouwman P. van Rooijen M. A. Pradere J. P. (2006). Autotaxin, a secreted lysophospholipase D, is essential for blood vessel formation during development. Mol. Cell Biol. 26 (13), 5015–5022. 10.1128/MCB.02419-05 16782887 PMC1489177

[B59] Wentzel P. Eriksson U. J. (2005). A diabetes-like environment increases malformation rate and diminishes prostaglandin E(2) in rat embryos: Reversal by administration of vitamin E and folic acid. Birth Defects Res. A Clin. Mol. Teratol. 73 (7), 506–511. 10.1002/bdra.20145 15959876

[B60] Wiznitzer A. Ayalon N. Hershkovitz R. Khamaisi M. Reece E. A. Trischler H. (1999). Lipoic acid prevention of neural tube defects in offspring of rats with streptozocin-induced diabetes. Am. J. Obstet. Gynecol. 180 (1), 188–193. 10.1016/s0002-9378(99)70173-0 9914602

[B61] Zabihi S. Eriksson U. J. Wentzel P. (2007). Folic acid supplementation affects ROS scavenging enzymes, enhances Vegf-A, and diminishes apoptotic state in yolk sacs of embryos of diabetic rats. Reprod. Toxicol. 23 (4), 486–498. 10.1016/j.reprotox.2007.03.007 17482424

[B62] Zhao J. Hakvoort T. B. M. Ruijter J. M. Jongejan A. Koster J. Swagemakers S. M. A. (2017). Maternal diabetes causes developmental delay and death in early-somite mouse embryos. Sci. Rep. 7 (1), 11714. 10.1038/s41598-017-11696-x 28916763 PMC5601907

[B63] Zohn I. E. Sarkar A. A. (2010). The visceral yolk sac endoderm provides for absorption of nutrients to the embryo during neurulation. Birth Defects Res. A Clin. Mol. Teratol. 88 (8), 593–600. 10.1002/bdra.20705 20672346

